# Consumption of Low-Calorie Sweetened Beverages Compared to Water Is Associated with Reduced Intake of Carbohydrates and Sugar, with No Adverse Relationships to Glycemic Responses: Results from the 2001–2012 National Health and Nutrition Examination Surveys

**DOI:** 10.3390/nu9090928

**Published:** 2017-08-24

**Authors:** Marge Leahy, Joseph C. Ratliff, Claudia S. Riedt, Victor L. Fulgoni

**Affiliations:** 1 Food, Nutrition and Policy Consultant, Fort Myers Beach, FL 33931, USA; margeleahy@yahoo.com; 2 Dr. Pepper Snapple Group, 5301 Legacy Drive, Plano, TX 75024, USA; joseph.ratliff@dpsg.com; 3 Nutrition Impact, LLC, 9725 D Drive North, Battle Creek, MI 49014, USA; vic3rd@aol.com

**Keywords:** diet drinks, beverages, low-calorie sweeteners, prediabetes, National Health and Nutrition Examination Survey

## Abstract

Although the 2015 Dietary Guidelines Advisory Committee concluded that there was moderate evidence that substituting sugar-containing sweeteners with low-calorie sweeteners (LCS) reduces calorie intake and weight, dietary recommendations encourage substituting only water for sugar-sweetened beverages during weight management. This cross-sectional study evaluated the relation of water and no- and low-calorie sweetened beverage (LCSB) intake with nutrient intakes and prediabetes criteria using data from the National Health and Nutrition Examination Survey (NHANES) 2001–2012 in 25,817 adults that were free of diabetes. Although linear trends were observed with both beverages, higher LCSB intake was associated with significantly lower consumption of carbohydrates (−9.1 g/day vs. −1.4 g/day), total sugars (−10.9 g/day vs. −2.2 g/day), and added sugars (−2.0 tsp eq vs. −0.8 tsp eq) than those associated with higher water intake. Higher intake of both beverages was significantly associated with lower insulin levels (*p* < 0.01); however, higher intake of LCSB was also associated with lower hemoglobin A1c (HbA1c) and lower homeostatic model assessment of insulin resistance (HOMA-IR) (*p* < 0.01). We observed lower odds ratios for elevated HbA1c (adjusted odds ratio [OR] 0.79, 95% CI 0.64–0.98), HOMA-IR (0.68, 0.53–0.87), and insulin levels (0.63, 0.49–0.80) in LCSB among the higher (2+ servings) intake group compared to the lowest (<1 serving) intake group. Contrary to conventional wisdom, LCSB consumption was associated with equal, if not better, dietary intake and glycemic response than water consumption. Although observational in nature, these results contribute to the growing body of evidence from human studies suggesting that in addition to water, LCSBs can also be sensible choices for reducing sugars and carbohydrate intake, with no adverse associations to measures of glycemic response.

## 1. Introduction

According to the National Health and Nutrition Examination Survey (NHANES) 2009–2010, approximately 20% of the US population consumes diet drinks on a given day [1]. No- and low-calorie-sweetened beverages (LCSBs), some of them referred to as artificially-sweetened beverages, are beverages sweetened with low-calorie sweeteners (LCS) or non-nutritive sweeteners, like aspartame, sucralose, stevia, or acesulfame potassium to provide sweet taste with fewer or no calories. LCSBs may assist in weight control when displacing full-calorie beverages in the diet, if the resulting reduction in energy intake is not subsequently compensated [2]. The American Diabetes Association, The American Heart Association, and The Academy of Nutrition and Dietetics position statements acknowledge that LCS may be used within a structured diet plan to replace added sugars intake, thereby promoting reduced energy intake, and weight control or weight loss [3,4]. Additionally, the 2015 Dietary Guidelines Advisory Committee concluded there was moderate evidence from randomized-controlled trials (RCTs) that substituting sugar-containing sweeteners with LCS can reduce calorie intake, body weight, and adiposity. However, only water was designated as a preferred zero-calorie beverage option that could be consumed to facilitate long-term weight maintenance or weight loss, and the use of LCSBs as a replacement was discouraged [5].

Although two recent meta-analyses of RCTs reported modest weight reduction when LCS was substituted in the diet [6,7], there has been reticence in recommending LCSBs as a weight control tool because some epidemiological studies have reported positive associations between LCSB consumption and weight-related outcomes [8,9]. The associations between LCSBs and weight-related outcomes noted in some observational studies have fueled speculation about purported mechanisms to explain this possible relationship. Though it has often been suggested that reverse causation might best explain an association [6,7,10], one alternative hypothesis is that LCSBs could contribute to weight gain if they increased the desire and appetite for sweet foods or encouraged additional energy intake [11].

Excess body weight can increase the risk of the development of prediabetes and diabetes. Prediabetes is a condition in which blood glucose levels or hemoglobin A1c (HbA1c) are higher than normal but not yet at the level for a diagnosis of diabetes [12]. Diabetes is associated with serious health complications and mortality; the rates of deaths from all causes were 50% higher among adults with diagnosed diabetes than among adults without diagnosed diabetes [13]. In 2011–2012, the estimated prevalence of diabetes was 12% to 14% and prevalence of prediabetes was 37% to 38% in the United States [14]. Without diet- and physical-activity-related lifestyle modifications to improve their health, 15% to 30% of individuals with prediabetes will develop type 2 diabetes within five years [13]. The American Diabetes Association recommends monitoring carbohydrate intake as a key strategy for achieving glycemic control [15]; choosing to drink LCSBs or water is one method to assist with moderating carbohydrate intake [4].

A recent systematic review of LCS consumption noted that the effect of LCS on glucose metabolism could not be established [16]. The studies reviewed tended to focus on the risk of development of diabetes or assessed individuals that were already diabetic. The present study adds to this body of knowledge by evaluating the associations of LCSB and water consumption patterns with nutrient intakes, prediabetes criteria, and other glycemic variables in healthy adults from NHANES (2001–2012).

## 2. Materials and Methods 

### 2.1. Subjects

Data from What We Eat In America (WWEIA) 2001–2012, the dietary intake component of the National Health and Nutrition Examination Survey (NHANES), were used to assess intake of LCSB and water [17]. NHANES is a continuous survey conducted by the National Center for Health Statistics (NCHS). The present analysis combined six NHANES datasets (NHANES 2001–2002, 2003–2004, 2005–2006, 2007–2008, 2009–2010, and 2011–2012). Adults age 19 years and older (*n* = 29,687) with reliable 24-h recall dietary interviews (one conducted in person and another via the telephone) were included in the analyses; these interviews were conducted using the best procedures available, namely, the United States Department of Agriculture’s (USDA) automated multiple-pass method [15]. Pregnant and/or lactating females and those with incomplete or unreliable 24-h recall data were excluded. Additionally, those subjects that reported they were told by a doctor that they had diabetes (*n* = 3877) were also excluded, leaving an analytical sample of 25,817. All participants provided written informed consent and the Research Ethics Review Board at the NCHS approved the survey protocol. The reporting of this study conforms to the STROBE statement [18].

### 2.2. Estimation of Intake 

Intakes of water were defined as drinking water using the response to NHANES question “Total plain water drank yesterday (gm)”. Thus, water includes “plain tap water, water from a drinking fountain, water from a water cooler, bottled water, and spring water [19]”. LCSB were defined as beverages with ≤6.7 calories/8oz and are listed in Table 1 (Table S1 provides the percentage of use of LCSB by age and gender groups) [20]. The individual usual intake of LCSB and water were determined using the two part model of the National Cancer Institute (NCI) method [21], and were used to separate subjects into those with <1, 1–2, and >2 servings of each beverage. Energy and nutrient intake were determined using the USDA Nutrient Database for Standard Reference Releases [22] in conjunction with the respective Food & Nutrient Database for Dietary Studies for each NHANES cycle participant [20]. The USDA MyPyramid Equivalents Database (MPED) and Food Patterns Equivalents Database (FPED) were used to calculate intake of MyPlate servings [23,24]. The MPED and FPED translate dietary recall data into equivalent servings of the MyPlate major food groups and corresponding subgroups. The number of MyPlate servings was aggregated over all foods consumed during the 24-h recall to calculate the MyPlate food group intakes per day. Energy and nutrient intakes based on LCSB and water, and serving levels within each beverage type, were estimated using dietary intake data.

### 2.3. Demographics

Characteristics defining the entire sample and those in each serving level within each beverage category were based on responses to standard NHANES questions (for age, gender, race/ethnicity, physical activity, poverty index ratio) or determined from measurements during the medical examination (body mass index).

### 2.4. Glycemic Variables

Fasting plasma glucose, serum insulin, and HbA1c were obtained from NHANES laboratory files [25]. Homeostasis model assessment: insulin resistance (HOMA-IR) was calculated as insulin (mU/L) × plasma glucose (mmol/L)/22.5 [26].

### 2.5. Statistical Analysis

Least square means (LSM) and standard errors (SE) were determined for energy intake and macronutrient intakes by LCSB and water serving levels via regression analyses. The data were adjusted for the complex sample design of NHANES using appropriate survey weights, strata, and primary sampling units. Dietary weights were used in all intake analyses. Covariates for the regression analyses were age, gender, ethnicity, poverty income ratio, physical activity level, current smoking status, alcohol intake, and energy intake (except for energy intake analyses). Regression analyses of glycemic variables included the covariates above and also BMI. Logistic regression was used within each beverage category to assess odds ratios (95th confidence levels) of exceeding certain levels of glycemic variables [glucose ≥ 5.55 mmol/L; insulin ≥ 90 pmol/L; HbA1c ≥ 5.7%; and HOMA-IR ≥ 4—the midpoint of moderate insulin resistance (3–5)] [27,28] with the covariates (including BMI) above; the <1 serving group within each beverage category was the reference value (odds ratio set at 1.00). Within each beverage category trend analyses were conducted to evaluate the impact of the increased number of servings; *p*-value was set at *p* < 0.01 to adjust for multiple comparisons to protect against type 1 error. To assess differences in trends across serving levels between beverage categories, we examined confidence intervals of trends; non-overlapping 99th percentiles confidence intervals were deemed significant. SAS 9 (SAS Institute, Cary, NC, USA) and SUDAAN 11 (RTI, Research Triangle Park, NC, USA) were used for all analyses.

## 3. Results

### 3.1. Demographics and Characteristics of LCSB and Water Consumers

Women were more likely to consume 1–2 servings per day of LCSBs than men, whereas men were more likely to consume 1–2 servings of waters per day than women (Table 2). Women represented a greater proportion of the consumers of 2+ servings for both beverage categories. For both LCSB and water consumption, vigorous physical activity was highest in the 2+ servings groups. The percentage of consumers with usual intakes of 2+ servings of LCSB was highest in adults aged 35 to 50, whereas usual intakes of 2+ servings of water remained consistent with age (Table S1).

### 3.2. Nutrient Intakes

There was no significant association between total energy intake and higher consumption of either LCSBs or water (Table 3). Although linear trends were observed with both beverages, higher LCSB intake was associated with significantly lower consumption of carbohydrates (−9.1 g/day vs. −1.4 g/day), total sugars (−10.9 g/day vs. −2.2 g/day), and added sugars (−2.0 tsp eq vs. −0.8 tsp eq) than those associated with higher water intake. Higher intake of both beverages was significantly associated with higher consumption of protein, total fat, MFA, and PUFA. Additionally, water consumption was linearly associated with higher intake of dietary fiber, while LCSB consumption was linearly associated with higher intake of SFA. These associations were consistent in both genders (Table S2). 

### 3.3. Prediabetes Criteria

Higher intake of both beverages was significantly associated with lower insulin levels (*p* < 0.01), while higher intake of LCSB was also associated with lower HbA1c and lower HOMA-IR (*p* < 0.01) (Table 4). In gender-specific analyses, similar trends were noticed but only in females was the association of lower insulin and HOMA-IR with a higher intake of LCSB significant (Table S3). There were lower odds ratios for higher insulin (adjusted odds ratio [OR]: 0.63, 95% CI: 0.49–0.80), HbA1c (OR: 0.79, 95% CI: 0.64–0.98) and HOMA-IR levels (OR; 0.68, 95% CI: 0.53–0.87) in LCSB in the higher (2+ servings) intake group as compared to the lowest (<1 serving) intake of LCSB (Table 5). None of the odds ratios for water intake reached significance, but there was a significant trend for lower odds ratios for elevated insulin levels with higher water intake; there was also a significant trend for lower odds ratios of elevated insulin, HbA1c, and HOMA-IR with higher LCSB intake. In gender-specific analyses, in general, the association of higher intake of LCSB and water with lower odds ratios of elevated glycemic variables was more evident in males than females (Table S4).

## 4. Discussion

Although the safety of LCS consumption has been confirmed on numerous occasions [4,5], the 2015 Dietary Guidelines recommend replacing sugar-sweetened beverages with water but not diet beverages sweetened with LCS. This study found that higher LCSB intake was associated with significantly lower consumption of carbohydrates and total and added sugars than those associated with higher water intake. The results of our analyses add to the body of evidence that consumption of LCSB is not associated with greater intake of sweets [29]. Dietary survey data from the UK [30] and the National Weight Control Registry [31] are in accordance with our results. Those surveys reported that LCSB consumers drink LCSBs to help control total calorie intake and that LCSB drinkers do not compensate for sugar or energy deficits by consuming more sugary food. Our findings differ from Piernas et al. [32] who examined dietary quality using Nielsen Homescan purchase data. They reported that, compared with nonconsumers, LCSB consumers had a higher percentage of energy intake from calorically-sweetened desserts. However, the strength of the conclusions that can be drawn from purchase studies is limited because they are not a true measure of consumption, as they neglect the consumption of unpackaged foods and foods eaten away from the home [30]. Consumption of LCSBs can provide sweetness and variety to the diet and aligns with the 2015 Dietary Guidelines’ recommendations to reduce sugar intake [5]. 

Higher intake of both beverage types was significantly associated with lower insulin levels, whereas higher intake of LCSB was also associated with lower HbA1c and lower HOMA-IR. These results suggest that higher LCSB consumption is not associated with adverse associations with prediabetes diagnostic markers or a glycemic response compared to plain water. Fagherazzi et al. [33] reported that both a high frequency and a longer consumption period of LCS in packages or tablets was independently associated with type 2 diabetes risk in a European prospective cohort of women, but that the risk was partially mediated by adiposity. These results differ from those found in our study in several ways. First, our study focused on LCS beverages which, according to 2009–2012 NHANES data, accounts for a much larger portion of LCS intake in adults than from packets [34], although there may be regional differences in LCS consumption patterns [35]. Second, that cohort study only investigated women, whereas we assessed both genders. The difference in study population is important because we report that the association of higher intake of LCSB (and water) with lower odds ratios of elevated glycemic variables was more evident in males than females. Finally, our study used prediabetes cutoffs instead of diabetes diagnoses. A recent review evaluated the evidence regarding a hypothetical effect of LCS on glucose metabolism and development of diabetes [16]. The authors concluded that the association between LCS intake and the development of type 2 diabetes is not clear and that an effect of LCS on glucose metabolism could not be established. Some observational studies have reported associations between LCS intake and the development of metabolic disease [16]; however, adiposity is a common confounding factor in observational prospective studies. The absence of adverse associations with glycemic response in our study suggest that LCSB consumption, which was associated with a lower risk of meeting prediabetes criteria in our study, may represent an additional beverage choice that could be utilized to help moderate sugar and carbohydrate intake. 

In this study, higher intake of either LCSBs or water was not significantly associated with higher total energy intake. A recent one year clinical trial found that LCSBs were a more effective tool than water to assist with weight loss and maintenance among regular users of non-nutritive sweeteners when used as part of a behavioral weight loss program [36,37]. An and McCaffrey [38] reported that total dietary water consumption was associated with reductions in total energy intake from sugar-sweetened beverages and discretionary foods. Phelan et al. [31] examined the dietary strategies employed by successful weight loss maintainers using the National Weight Control Registry. They found that successful weight loss maintainers consumed significantly more servings of diet soft drinks and water than a normal weight control group. One possible reason that we did not observe an association with lower total energy intake in this study could be because higher intake of both beverage types was significantly associated with higher consumption of protein, total fat, MFA, and PUFA. Additionally, higher LCSB consumption was linearly associated with higher intake of SFA. Mattes and Popkin [10] conducted a qualitative review of LCS consumption and potential effects on appetite, calorie intake, and body weight. Though studies were limited, they concluded that the use of LCS may result in a greater relative contribution of dietary fat to total energy intake but would not necessarily increase total calorie intake or body weight. Furthermore, knowledge of the use of LCS has been shown to result in energy compensation or even overcompensation in short-term trials; however, this effect is diminished with longer term use [36]. These findings underscore a need for education on total dietary choices. While dietary guidance regarding reducing added sugar can be an important tool for individuals to achieve recommended nutrient intakes within their calorie needs, guidance is also important regarding total calorie intake, fat quality, and avoiding compensation with other energy-rich foods. 

The strengths of this study include the use of a large, nationally-representative sample of adults by combining several NHANES datasets, adjusting for several covariates, and adjusting our statistical analyses for multiple comparisons to improve the reliability of the estimates. To our knowledge, this is the first study to analyze associations between LCSB and water intake with prediabetes criteria. 

There are some limitations that also should be taken into account. Cross-sectional data cannot be used to establish causation. Additionally, this study used self-reported dietary 24-h intakes which may not represent typical dietary consumption. The accuracy and currency of dietary data have limitations, thus potentially contributing to inaccuracies in estimating nutrient intakes. However, the US Department of Agriculture Automated Multiple-Pass Method, used in collecting 24-h diet recalls in the WWEIA of the NHANES, reduces bias in the collection of energy intakes [39]. This study did not evaluate any potential overlap between groups or the intake of LCS from foods; high water consumers may also have high LCS or LCSB consumption as well. Although several covariates were adjusted for in the analyses, some residual confounding may still exist. Other unadjusted factors that may also affect prediabetes risk include family history; other diseases or conditions, like polycystic ovary syndrome or hypertension; and certain medications, like glucocorticoids or atypical antipsychotics. Measures of glycemic control in this study represent a single fasting measurement; it would be helpful for future research to evaluate long-term relationships through clinical trials that also assess the post-prandial response and evaluate sweeteners individually. Although statistically significant differences were found as indicated, practical implications to dietary intakes and health outcomes have not been established.

## 5. Conclusions

Consumption of LCSBs is a common method to assist in weight control. Although cross-sectional in nature, our results contribute to the growing body of evidence from human studies that do not support the hypothesis that consumption of LCS is associated with greater intakes of sweets and total energy. Higher LCSB consumption was associated with significantly lower intake of carbohydrates and total and added sugar. Higher consumption of LCSBs was also associated with lower odds ratios for elevated levels of several prediabetes markers. Overall, these findings suggest that LCSBs, in addition to water, may also be sensible beverage choices to help moderate sugar and carbohydrate intake.

## Figures and Tables

**Table 1:** Prevalence of consumption of no- and low-calorie beverages in adults 19+ NHANES 2001–2012 ^1^.

Food Code	Description	Kcal/8oz	Consumption Occasions	Number of Consumers
92410320	Soft drink, cola, diet	4.5	6199	3144
92410350	Soft drink, cola, decaffeinated, diet	2.5	2306	1396
92410520	Soft drink, fruit flavored, diet, caffeine free	0.0	1206	803
92552010	Fruit flavored drink, powdered, reconstituted, diet	3.5	796	461
92301080	Tea, NS as to type, presweetened with low calorie sweetener	6.1	595	398
92410560	Soft drink, fruit flavored, caffeine, diet	0.0	578	315
92410370	Soft drink, pepper type, diet	4.5	544	339
92302300	Tea, leaf, presweetened with low calorie sweetener	6.0	311	202
92305090	Tea, iced, instant, black, pre-sweetened with low calorie sweetener	4.7	290	185
92410720	Soft drink, root beer, diet	0.0	214	166
94100200	Water, bottled, sweetened with low calorie sweetener	2.4	175	125
92550620	Fruit flavored drink, diet	7.1	167	125
92410620	Soft drink, ginger ale, diet	0.0	163	112
92410400	Soft drink, pepper type, decaffeinated, diet	2.5	158	120
92410250	Carbonated water, sweetened with low-calorie or no-calorie sweetener	0.0	134	95
92302700	Tea, leaf, decaffeinated, presweetened with low calorie sweetener	6.0	123	72
92305110	Tea, iced, instant, black, decaffeinated, pre-sweetened with low calorie sweetener	5.6	118	83
92550040	Fruit juice drink, diet	5.3	103	82
92301180	Tea, NS as to type, decaffeinated, presweetened with low calorie sweetener	6.0	65	50
92741000	Fruit-flavored drink, non-carbonated, made from low calorie powdered mix	2.6	64	47
92411610	Soft drink, cola, fruit or vanilla flavored, diet	4.6	61	48
92541040	Lemonade-flavored drink, made from powdered mix, low calorie	4.9	55	32
92306030	Tea, herbal, presweetened with low calorie sweetener	6.0	49	41
92520910	Lemonade, low calorie	4.8	41	37
92410420	Soft drink, cream soda, diet	0.0	20	16
92400100	Soft drink, NFS, diet	3.7	10	8
92565200	Powerade Zero sports drink, low calorie	0.0	3	2
95312500	Mountain Dew AMP Energy Drink, sugar-free	4.6	3	3
92650210	Mountain Dew AMP Energy Drink, sugar-free	4.7	2	2
92650805	Vault Zero Energy drink	2.5	2	1
92410820	Soft drink, chocolate flavored, diet	3.9	1	1
94220200	Glaceau Water, low calorie	2.4	1	1
95312800	Vault Zero Energy Drink	2.0	1	1

^1^ Individual usual intake was determined for each subject using the National Cancer Institute (NCI) method. Based on the individual intake, the percentage consumers of low-calorie sweetened beverages (LCSBs) and water was estimated by age group, gender, and servings categories.

**Table 2:** Demographic characteristics associated with no- and low-calorie sweetened beverage consumption in adults 19+ years of age: NHANES 2001–2012.

		No- and Low-Calorie Sweetened Beverages			Water		
	All	<1 Serving	1–2 Servings	2+ Servings	<1 Serving	1–2 Servings	2+ Serving
Gender							
% Female	50.7 ± 0.3	49.8 ± 0.4	54.7 ± 1.3	54.6 ± 1.6	54.0 ± 1.3	45.2 ± 1.4	51.1 ± 0.4
Ethnicity							
% Mexican American	7.9 ± 0.6	8.7 ± 0.7	5.7 ± 0.8	3.2 ± 0.6	7.8 ± 0.8	8.8 ± 0.9	7.7 ± 0.6
% Non-Hispanic black	10.9 ± 0.7	12.5 ± 0.8	6.3 ± 0.7	2.3 ± 0.3	11.5 ± 1.1	13.7 ± 1.1	10.3 ± 0.7
% Non-Hispanic white	71.3 ± 1.3	67.8 ± 1.4	81.3 ± 1.3	89.0 ± 1.1	72.7 ± 1.8	67.7 ± 1.9	71.6 ± 1.3
Physical Activity							
% Sedentary	25.9 ± 0.6	27.2 ± 0.6	20.7 ± 1.3	21.0 ± 1.3	37.6 ± 1.4	29.9 ± 1.1	23.7 ± 0.6
% Moderate	34.6 ± 0.5	34.0 ± 0.5	37.3 ± 1.4	36.4 ± 1.5	34.3 ± 1.3	34.6 ± 1.1	34.6 ± 0.6
% Vigorous	39.5 ± 0.8	38.8 ± 0.8	42.1 ± 1.5	42.5 ± 1.6	28.1 ± 1.3	35.6 ± 1.2	41.7 ± 0.8
Poverty Index Ratio (PIR)							
% PIR ≤ 1.3	21.5 ± 0.7	24.2 ± 0.8	12.2 ± 0.9	9.4 ± 0.8	28.9 ± 1.3	27.1 ± 1.4	19.5 ± 0.7
% 1.3 < PIR < 1.85	10.1 ± 0.3	10.9 ± 0.3	7.7 ± 0.9	6.0 ± 0.7	11.1 ± 0.8	10.6 ± 0.7	9.9 ± 0.3
% PIR > 1.85	68.5 ± 0.8	65.0 ± 0.9	80.1 ± 1.3	84.7 ± 1.1	60.0 ± 1.5	62.4 ± 1.6	70.6 ± 0.8
Other							
BMI (kg/m^2^)	28.0 ± 0.1	27.6 ± 0.1	29.3 ± 0.2	30.1 ± 0.2	27.9 ± 0.2	28.0 ± 0.2	28.1 ± 0.1
Age (years)	45.2 ± 0.3	44.8 ± 0.3	47.2 ± 0.4	46.0 ± 0.4	45.5 ± 0.4	45.3 ± 0.5	45.1 ± 0.3
*n*	25,815	22,027	2116	1672	2949	3444	19,422
Weighted *n*	191,034,976	153,129,025	19,016,197	18,889,754	20,941,619	23,173,180	146,920,177

Individual usual intake was determined for each subject using the NCI method. Based on the individual intake, the percentage of consumers of LCSB and water was estimated by age group, gender, and servings categories. Values are mean ± SE. A serving is defined as 8 fl oz.

**Table 3:** Associations between beverage consumption, energy, and macronutrient intakes in adults 19+ years of age: NHANES 2001–2012.

Variables	No- and Low-Calorie Sweetened Beverages				Water				LCSB vs. Water Trend
	<1 Serving	1–2 Servings	>2 Servings	Linear Trend	<1 Serving	1–2 Servings	>2 Servings	Linear Trend	Linear Trend
Energy (kcal)	2134 ± 10	2038 ± 23	2080 ± 23	0.037	2170 ± 24	2136 ± 23	2114 ± 11	0.473	NS
Carbohydrate (g)	272 ± 1	257 ± 2	244 ± 2	<0.001	276 ± 2	273 ± 2	267 ± 1	<0.001	*
Total sugar (g)	125 ± 1	106 ± 1	92 ± 2	<0.001	137 ± 2	129 ± 2	118 ± 1	<0.001	*
Added sugar (tsp eq)	19.7 ± 0.2	15.8 ± 0.4	13.7 ± 0.5	<0.001	23.8 ± 0.5	21.4 ± 0.4	17.9 ± 0.2	<0.001	*
Dietary fiber (g)	16.5 ± 0.2	16.8 ± 0.3	16.2 ± 0.3	0.334	14.4 ± 0.2	15.1 ± 0.2	17.1 ± 0.1	<0.001	*
Protein (g)	82.3 ± 0.4	86.0 ± 0.8	87.9 ± 1.0	<0.001	79.7 ± 0.7	80.3 ± 0.8	87.9 ± 1.0	<0.001	*
Total fat (g)	78.1 ± 0.3	82.9 ± 0.8	87.3 ± 0.9	<0.001	77.1 ± 0.7	78.0 ± 0.6	79.5 ± 0.3	<0.001	*
Total MFA (g)	28.8 ± 0.1	30.6 ± 0.3	32.3 ± 0.4	<0.001	28.5 ± 0.3	28.8 ± 0.2	29.3 ± 0.1	0.001	*
Total PUFA (g)	17.3 ± 0.1	18.6 ± 0.3	19.9 ± 0.3	<0.001	16.5 ± 0.3	17.4 ± 0.3	17.7 ± 0.1	0.001	*
Total SFA (g)	25.1 ± 0.1	26.4 ± 0.3	27.5 ± 0.3	<0.001	25.2 ± 0.3	25.1 ± 0.2	25.4 ± 0.1	0.206	*

Individual usual intake was determined for each subject using the NCI method. Based on the individual intake, the percentage consumers of LCSB and water was estimated by age group, gender, and servings categories. Values are least square mean ± SE from regression models with age, gender, ethnicity, current smoking (Y/N), poverty income ratio, physical activity level (sedentary, moderate, vigorous based on responses to questions), and alcohol intake. Energy intake was added as a covariate for macronutrient intakes. NS: Indicates 99th percentile confidence intervals of beta coefficients for servings for LCSB and water overlap. * Indicates 99th percentile confidence intervals of beta coefficients for servings for LCSB and water do not overlap. Abbreviations: LCSB, no-and low-calorie sweetened beverages; MFA, monounsaturated fatty acids; PUFA, polyunsaturated fatty acids; SFA, saturated fatty acids.

**Table 4:** Associations between beverage consumption and measures of glycemic control in adults 19+ years of age: NHANES 2001–2012.

Variables	No- and Low-Calorie Sweetened Beverages				Water				LCSB vs. Water Trend
	<1 Serving	1–2 Servings	>2 Servings	Linear Trend	<1 Serving	1–2 Servings	>2 Servings	Linear Trend	Linear Trend
Glucose (mmol/L)	5.57 ± 0.02	5.56 ± 0.03	5.51 ± 0.03	0.046	5.57 ± 0.03	5.56 ± 0.02	5.56 ± 0.02	0.145	NS
Insulin (pmol/L)	73.2 ± 1.0	70.5 ± 2.5	65.7 ± 2.0	<0.001	75.1 ± 2.0	73.7 ± 1.7	72.1 ± 1.1	0.009	NS
HbA1c (%)	5.50 ± 0.01	5.49 ± 0.01	5.46 ± 0.01	0.005	5.49 ± 0.01	5.50 ± 0.01	5.50 ± 0.01	0.298	NS
HOMA-IR	3.13 ± 0.05	3.04 ± 0.13	2.79 ± 0.09	<0.001	3.20 ± 0.10	3.11 ± 0.08	3.09 ± 0.05	0.061	NS

Individual usual intake was determined for each subject using the NCI method. Based on the individual intake, the percentage consumers of LCSB and water was estimated by age group, gender, and servings categories. Values are least square mean ± SE from regression models with age, gender, ethnicity, current smoking (Y/N), poverty income ratio, physical activity level (sedentary, moderate, vigorous based on responses to questions), alcohol intake, and body mass index. NS: Indicates 99th percentile confidence intervals of beta coefficients for servings for LCSB and water overlap. Insulin resistance calculated as insulin (mU/L) × glucose (mmol/L)/22.5. Abbreviations: LCSB, no-and low-calorie sweetened beverages; HOMA-IR, homeostasis model assessment of insulin resistance.

**Table 5:** Associations between beverage consumption and measures with odds ratios of risk for glycemic variables in adults 19+ years of age: NHANES 2001–2012.

Variables	No- and Low-Calorie Sweetened Beverages				Water			
	<1 Serving	1–2 Servings	>2 Servings	Linear Trend	<1 Serving	1–2 Servings	>2 Servings	Linear Trend
Glucose ≥ 5.55 mmol/L	1.00	1.13 (0.93, 1.37)	0.95 (0.79, 1.14)	0.487	1.00	1.12 (0.91, 1.39)	1.01 (0.84, 1.21)	0.498
Insulin ≥ 90 pmol/L	1.00	0.74 * (0.59, 0.94)	0.63 * (0.49, 0.80)	<0.001	1.00	0.80 (0.61, 1.04)	0.79 (0.63, 1.01)	0.001
HbA1c ≥ 5.7%	1.00	0.97 (0.83, 1.13)	0.79 * (0.64, 0.98)	0.002	1.00	1.20 (0.98, 1.45)	1.10 (0.95, 1.28)	0.431
HOMA-IR ≥ 4.0	1.00	0.87 (0.69, 1.10)	0.68 * (0.53, 0.87)	0.009	1.00	0.84 (0.65, 1.08)	0.80 (0.64, 1.00)	<0.001

Individual usual intake was determined for each subject using the NCI method. Based on the individual intake, the percentage consumers of LCSB and water was estimated by age group, gender, and servings categories. Values are odds ratios (95th percentile confidence limits) with <1 serving within each beverage set as reference value with odds ratio of 1.00 from logistics regression models with age, gender, ethnicity, current smoking (Y/N), poverty income ratio, physical activity level (sedentary, moderate, vigorous based on responses to questions), alcohol intake, and body mass index. Insulin resistance calculated as insulin (mU/L) × glucose (mmol/L)/22.5. Abbreviations: LCSB, no-and low-calorie sweetened beverages; HOMA-IR, homeostasis model assessment of insulin resistance.

## Supplementary Materials

The following are available online at www.mdpi.com/2072-6643/9/9/928/s1, Table S1: Percentage of Consumers’ Usual intakes by Age and Gender, Adults 19+ years of age: NHANES 2001–2012, Table S2: Associations between beverage consumption and energy and macronutrient intakes in males and females 19+ years of age: NHANES 2001–2012, Table S3: Associations between beverage consumption and measures of glycemic control in adult females and males 19+ years of age: NHANES 2001–2012, Table S4: Associations between beverage consumption and measures with odds ratios of risk for glycemic variables in adult females and males 19+ years of age: NHANES 2001–2012.
