# Supplementary material for: Consumption of Low-Calorie Sweetened Beverages Compared to Water Is Associated with Reduced Intake of Carbohydrates and Sugar, with No Adverse Relationships to Glycemic Responses: Results from the 2001–2012 National Health and Nutrition Examination Surveys
Source: Nutrients. 2017 Aug 24;9(9):928. doi: 10.3390/nu9090928 (PMC5622688; doi:10.3390/nu9090928)
Supplement: Supplementary file 1 [file nutrients-09-00928-s001.zip › nutrients-216749-supplementary.pdf]

nutrients

Table S1. Percentage of consumers' usual intakes by age and gender, adults 19+ years of age: NHANES 2001–2012

Group	Age min.	Age max	Gender	N	< 1 Serving		1-2 Servings		2+ Servings	
					%	SE	%	SE	%	SE
Water	19	34	All	7,915	10.8	0.6	12.0	0.6	77.2	0.9
Water	19	34	Male	4,206	7.4	0.6	13.5	0.8	79.1	1.0
Water	19	34	Female	3,709	14.5	0.9	10.4	0.7	75.1	1.2
Water	35	50	All	7,232	10.1	0.5	12.4	0.6	77.4	0.8
Water	35	50	Male	3,598	8.9	0.7	14.5	0.8	76.6	1.1
Water	35	50	Female	3,634	11.4	0.7	10.4	0.7	78.2	1.0
Water	19	50	All	15,147	10.4	0.4	12.2	0.5	77.3	0.7
Water	19	50	Male	7,804	8.2	0.5	14.0	0.6	77.8	0.8
Water	19	50	Female	7,343	12.8	0.6	10.4	0.6	76.8	0.9
Water	51	99	All	10,667	11.9	0.5	11.9	0.5	76.2	0.6
Water	51	99	Male	5,285	14.3	0.8	12.5	0.9	73.2	1.0
Water	51	99	Female	5,382	9.8	0.6	11.4	0.6	78.7	0.8
Water	19	99	All	25,814	11.0	0.4	12.1	0.4	76.9	0.6
Water	19	99	Male	13,089	10.2	0.4	13.5	0.5	76.3	0.7
Water	19	99	Female	12,725	11.7	0.5	10.8	0.5	77.5	0.7
LCSB	19	34	All	7,915	86.8	0.6	7.3	0.5	5.9	0.4
LCSB	19	34	Male	4,206	89.7	0.7	5.7	0.6	4.6	0.5
LCSB	19	34	Female	3,709	83.5	1.0	9.2	0.7	7.4	0.7
LCSB	35	50	All	7,232	74.7	1.0	11.3	0.5	13.9	0.7
LCSB	35	50	Male	3,598	76.0	1.1	10.9	0.7	13.1	0.9
LCSB	35	50	Female	3,634	73.5	1.2	11.8	0.8	14.8	1.0
LCSB	19	50	All	15,147	80.6	0.7	9.4	0.4	10.0	0.5
LCSB	19	50	Male	7,804	82.9	0.8	8.3	0.5	8.8	0.6
LCSB	19	50	Female	7,343	78.2	0.9	10.5	0.5	11.3	0.7
LCSB	51	99	All	10,667	79.3	0.6	11.0	0.5	9.7	0.5
LCSB	51	99	Male	5,285	79.4	0.8	10.9	0.6	9.7	0.7
LCSB	51	99	Female	5,382	79.3	0.9	11.0	0.7	9.7	0.6
LCSB	19	99	All	25,814	80.2	0.5	10.0	0.3	9.9	0.4
LCSB	19	99	Male	13,089	81.7	0.6	9.1	0.4	9.1	0.5
LCSB	19	99	Female	12,725	78.6	0.7	10.7	0.4	10.6	0.5

Individual usual intake was determined for each subject using the NCI method. Based on individual intake, the percentage consumers of LCSB and Water was estimated by age group, gender, and servings categories. Abbreviations: LCSB, no-and low-calorie sweetened beverages

Table S2. Associations between beverage consumption and energy and macronutrient intakes in males and females 19+ years of age: NHANES 2001–2012

	**No- and Low-Calorie Sweetened beverages**				**Water**				**LCSB vs Water Trend**
Variables	< 1 serving	1-2 Servings	2+ Servings	Linear trend	< 1 Serving	1-2 Servings	2+ Servings	Linear trend	Linear trend
				Females					
Energy (kcal)	1784 ± 12	1704 ± 25	1730 ± 31	0.081	1831 ± 26	1776 ± 30	1766 ± 13	0.103	NS
Carbohydrate (g)	231 ± 1	219 ± 2	207 ± 2	<0.001	237 ± 2	230 ± 2	227 ± 1	<0.001	*
Total sugar (g)	108 ± 1	91 ± 2	79 ± 2	<0.001	119 ± 3	109 ± 2	102 ± 1	<0.001	*
Added sugar (tsp eq)	16.2 ± 0.2	13.0 ± 0.5	11.1 ± 0.5	<0.001	20.2 ± 0.6	17.5 ± 0.4	14.8 ± 0.2	<0.001	*
Dietary fiber (g)	14.9 ± 0.2	15.2 ± 0.3	14.2 ± 0.3	0.065	13.1 ± 0.3	13.8 ± 0.3	15.3 ± 0.2	<0.001	*
Protein (g)	67.6 ± 0.4	70.0 ± 0.9	72.2 ± 1.0	<0.001	64.5 ± 0.8	66.2 ± 0.9	68.9 ± 0.4	<0.001	NS
Total fat (g)	65.6± 0.3	69.6 ± 0.9	73.4 ± 0.9	<0.001	64.3 ± 1.0	66.2 ± 0.8	66.7 ± 0.3	0.148	*
Total MFA (g)	23.9 ± 0.2	25.2 ± 0.4	26.9 ± 0.4	<0.001	23.5 ± 0.4	24.0 ± 0.3	24.3 ± 0.2	0.072	*
Total PUFA (g)	15.0 ± 0.1	16.2 ± 0.3	16.9 ± 0.4	<0.001	14.1 ± 0.3	15.1 ± 0.4	15.3 ± 0.1	0.044	*
Total SFA (g)	20.9 ± 0.1	22.1 ± 0.4	23.2 ± 0.4	<0.001	20.8 ± 0.4	21.0 ± 0.3	21.2 ± 0.1	0.792	*
				Males					
Energy (kcal)	2454 ± 16	2362 ± 40	2426 ± 45	0.667	2514 ± 42	2467 ± 35	2435 ± 18	0.221	NS
Carbohydrate (g)	311 ± 1	292 ± 3	279 ± 4	<0.001	315 ± 3	314 ± 2	306 ± 1	<0.001	*
Total sugar (g)	142 ± 1	119 ± 3	104 ± 4	<0.001	154 ± 3	147 ± 2	134 ± 1	<0.001	*
Added sugar (tsp eq)	22.9 ± 0.3	18.5 ± 0.7	16.0 ± 0.8	<0.001	27.3 ± 0.8	25.1 ± 0.6	20.9 ± 0.3	<0.001	*
Dietary fiber (g)	18.1 ± 0.2	18.4 ± 0.4	18.2 ± 0.5	0.907	15.5 ± 0.3	16.6 ± 0.3	18.9 ± 0.2	<0.001	*
Protein (g)	96.4 ± 0.6	102 ± 1	103 ± 1	<0.001	94.7 ± 1.2	94.9 ± 1.1	98.2 ± 0.6	<0.001	*
Total fat (g)	90.4 ± 0.4	96.1 ± 1.2	101 ± 1	<0.001	89.3 ± 1.2	89.9 ± 0.8	92.1 ± 0.5	<0.001	*
Total MFA (g)	33.7 ± 0.3	35.9 ± 0.5	37.6 ± 0.6	<0.001	33.3 ± 0.5	33.5 ± 0.4	34.3 ± 0.2	0.005	*
Total PUFA (g)	19.5 ± 0.2	21.1 ± 0.5	22.9 ± 0.5	<0.001	18.7 ± 0.4	19.4 ± 0.3	20.1 ± 0.2	0.002	*
Total SFA (g)	29.2 ± 0.2	30.8 ± 0.6	31.8 ± 0.5	<0.001	29.6 ± 0.5	29.2 ± 0.4	29.5 ± 0.2	0.126	*

Individual usual intake was determined for each subject using the NCI method. Based on individual intake, the percentage consumers of LCSB and Water was estimated by age group, gender, and servings categories. Values are least square mean ± SE from regression models with age, gender, ethnicity, current smoking (Y/N), poverty income ratio, physical activity level (sedentary, moderate, vigorous based on responses to questions), and alcohol intake. Energy intake was added as a covariate for macronutrient intakes. NS: Indicates 99th percentile confidence intervals of beta coefficients for servings for LCSB and Water overlap. * Indicates 99th percentile confidence intervals of beta coefficients for servings for LCSB and Water do not overlap. Abbreviations: LCSB, no-and low-calorie sweetened beverages; MFA, monounsaturated fatty acids; PUFA, polyunsaturated fatty acids; SFA, saturated fatty acids.

Table S3. Associations between beverage consumption and measures of glycemic control in adult males and females 19+ years of age: NHANES 2001–2012

**Variables**	**No- and Low-Calorie Sweetened Beverages**				**Water**				**LCSB vs Water trend**
	< 1 Serving	1-2 Servings	> 2 Servings	Linear trend	< 1 Serving	1-2 Servings	> 2 Servings	Linear Trend	Linear trend
Females									
Glucose (mmol/L)	5.45 ± 0.02	5.39 ± 0.04	5.36 ± 0.04	0.010	5.43 ± 0.03	5.41 ± 0.03	5.43 ± 0.02	0.924	NS
Insulin (pmol/L)	70.9 ± 1.1	68.0 ± 2.2	63.1 ± 2.2	<0.001	72.7 ± 2.5	68.9 ± 1.7	70.0 ± 1.2	0.074	NS
HbA1c (%)	5.48 ± 0.01	5.48 ± 0.02	5.45 ± 0.02	0.081	5.46 ± 0.01	5.47 ± 0.01	5.48 ± 0.01	0.172	NS
HOMA-IR	2.96 ± 0.06	2.81 ± 0.11	2.58 ± 0.11	<0.001	3.00 ± 0.11	2.83 ± 0.08	2.93 ± 0.06	0.210	NS
Males									
Glucose (mmol/L)	5.69 ± 0.02	5.72 ± 0.05	5.66 ± 0.04	0.562	5.71 ± 0.04	5.71 ± 0.03	5.68 ± 0.02	0.071	NS
Insulin (pmol/L)	76.4 ± 1.7	72.6 ± 4.6	67.8 ± 3.4	0.013	77.7 ± 3.1	78.7 ± 2.8	74.7 ± 1.7	0.035	NS
HbA1c (%)	5.52 ± 0.01	5.51 ± 0.02	5.47 ± 0.02	0.041	5.51 ± 0.02	5.53 ± 0.02	5.51 ± 0.01	0.800	NS
HOMA-IR	3.34 ± 0.08	3.25 ± 0.23	2.96 ± 0.15	0.040	3.42 ± 0.15	3.41 ± 0.12	3.28 ± 0.08	0.107	NS

Individual usual intake was determined for each subject using the NCI method. Based on the individual intake, the percentage consumers of LCSB and Water was estimated by age group, gender, and servings categories. Values are least square mean ± SE from regression models with age, gender, ethnicity, current smoking (Y/N), poverty income ratio, physical activity level (sedentary, moderate, vigorous based on responses to questions), alcohol intake, body mass index. NS: Indicates 99th percentile confidence intervals of beta coefficients for servings for LCSB and Water overlap Insulin resistance calculated as insulin (mU/L) × glucose (mmol/L)/22.5. Abbreviations: LCSB, no-and low-calorie sweetened beverages; HOMA-IR, homeostasis model assessment of insulin resistance.

Table S4. Associations between beverage consumption and measures with odds ratios of risk for glycemic variables in adult males and females 19+ years of age: NHANES 2001–2012

Variables	No- and Low-Calorie Sweetened Beverages				Water			
	< 1 Serving	1-2 Servings	> 2 Servings	Linear trend	< 1 Serving	1-2 Servings	> 2 Servings	Linear Trend
				Females				
Glucose ≥ 5.55 mmol/L	1.00	1.04 (0.78, 1.40)	0.86 (0.64, 1.14)	0.424	1.00	1.04 (0.74, 1.46)	1.05 (0.82, 1.35)	0.980
Insulin ≥ 90 pmol/L	1.00	0.89 (0.63, 1.25)	0.58* (0.41, 0.84)	0.045	1.00	0.82 (0.55, 1.21)	0.94 (0.69, 1.26)	0.643
HbA1c ≥ 5.7 %	1.00	1.03 (0.82, 1.28)	0.87 (0.63, 1.20)	0.232	1.00	1.18 (0.87, 1.60)	1.24 (0.97, 1.58)	0.620
HOMA-IR ≥ 4.0	1.00	1.00 (0.70, 1.44)	0.60* (0.41, 0.89)	0.092	1.00	0.71 (0.47, 1.08)	1.02 (0.74, 1.39)	0.702
				Males				
Glucose ≥ 5.55 mmol/L	1.00	1.23 (0.94, 1.61)	1.07 (0.86, 1.34)	0.988	1.00	1.13 (0.83, 1.54)	0.95 (0.74, 1.20)	0.262
Insulin ≥ 90 pmol/L	1.00	0.61* (0.45, 0.82)	0.65* (0.45, 0.94)	0.005	1.00	0.76 (0.51, 1.14)	0.67* (0.47, 0.95)	<0.001
HbA1c ≥ 5.7 %	1.00	0.92 (0.71, 1.18)	0.70* (0.50, 0.98)	0.003	1.00	1.15 (0.88, 1.50)	0.95 (0.78, 1.15)	0.782
HOMA-IR ≥ 4.0	1.00	0.74* (0.57, 0.95)	0.73 (0.52, 1.01)	0.032	1.00	0.89 (0.63, 1.25)	0.65* (0.48, 0.87)	<0.001

Individual usual intake was determined for each subject using the NCI method. Based on the individual intake, the percentage consumers of LCSB and Water was estimated by age group, gender, and servings categories. Values are odds ratios (95th percentile confidence limits) with < 1 serving within each beverage set as reference value with odds ratio of 1.00 from logistics regression models with age, gender, ethnicity, current smoking (Y/N), poverty income ratio, physical activity level (sedentary, moderate, vigorous based on responses to questions), alcohol intake, and body mass index. Insulin resistance calculated as insulin (mU/L) X glucose (mmol/L)/22.5. Abbreviations: LCSB, no-and low-calorie sweetened beverages; HOMA-IR, homeostasis model assessment of insulin resistance
